# Vitamin D levels in non-pregnant women with a history of recurrent pregnancy loss with and without autoantibodies

**DOI:** 10.22088/cjim.15.2.266

**Published:** 2024

**Authors:** Tooba Farazmand, Raheleh Rahbarian, Mitra Jalali, Amirali Ghahremani, Abdollah Razi, Hasan Namdar Ahmadabad

**Affiliations:** 1 Department of Gynecology, School of Medicine, North Khorasan University of Medical Sciences, Bojnurd, Iran; 2 Department of Biology, Payame Noor University, Tehran, Iran; 3 Department of Internal Medicine, North Khorasan University of Medical Sciences, Bojnurd, Iran; 4 Department of Urology, Imam Ali Hospital, North Khorasan University of Medical Sciences, Bojnurd Iran; 5 Vector-borne Diseases Research Center North Khorasan University of Medical Sciences; 6 Department of Pathobiology and Laboratory Sciences, North Khorasan University of Medical Sciences, Bojnurd, Iran

**Keywords:** Vitamin D, Recurrent pregnancy loss, antinuclear antibody, Antiphospholipid antibody, Anti-thyroid peroxidase

## Abstract

**Background:**

The objective of this study was to compare the levels of vitamin D in non-pregnant women with a history of recurrent pregnancy loss (RPL) who were seropositive or seronegative for autoantibodies (autoAbs).

**Methods:**

The study examined 58 RPL patients with autoAbs (ANA, anti-TPO, or APAs), 34 RPL patients without autoAbs, and 58 healthy women with prior successful pregnancies and without autoantibodies. The levels of 25 (OH) D were measured using the sandwich ELISA technique.

**Results:**

Our results showed insufficient serum 25(OH) D levels in study groups, with significantly lower levels observed in RPL patients with or without autoAbs compared to healthy women (P=0.0006). In addition, RPL patients with autoAbs had significantly lower 25(OH) D levels compared to RPL patients without autoAbs. We also found that serum levels of 25(OH) D in RPL patients with autoAbs were significantly lower than in RPL patients without autoAbs (20.51 ± 1.15 ng/ml Vs. 23.69 ± 0.74 ng/ml, *P*=0.0356). Further analysis indicated that RPL patients who were positive for ANA, and APAs, except anti-TPO, had significantly lower than 25(OH)D serum levels than RPL patients without autoAbs.

**Conclusion:**

These findings suggest that RPL patients, especially those with APAs or ANA, have lower vitamin D levels compared to healthy women. This may indicate a link between maternal immune dysregulation due to vitamin D deficiency and the presence of autoantibodies in RPL.

Recurrent pregnancy loss (RPL), also known as recurrent spontaneous abortion, recurrent miscarriage, or habitual abortion, is delineated as the incidence of three or more consecutive spontaneous abortions. This condition impacts nearly 1% to 2% of pregnant women (1, 2). The categorization of RPL encompasses primary and secondary types. Primary RPL refers to the loss of pregnancy before 24 weeks gestation without any prior ongoing pregnancies. Conversely, secondary RPL pertains to the occurrence of pregnancy loss after one or more previous pregnancies have progressed beyond 24 weeks (2, 3). Factors implicated in the etiology of RPL are advanced female age, previous abortion, chromosomal abnormalities, certain uterine anatomic abnormalities, endocrine disorders, untreated hypothyroidism, uncontrolled diabetes mellitus, infections, antiphospholipid antibody syndrome (APS), immunologic abnormalities, environmental factors (1, 4, 2). Notwithstanding numerous established etiologic variables associated with recurrent pregnancy loss (RPL), approximately fifty percent of all cases remain unexplained (5, 3, 6). Among several immunological factors associated with RPL, several studies suggest that autoimmune factors can cause fetal rejection in 30% of women with RPL (7, 8). 

Autoreactive antibodies, specifically antinuclear antibodies (ANA), anti-thyroid antibodies (ATA), and antiphospholipid antibodies (APAs), including lupus anticoagulant (LA), anti-cardiolipin (aCL), anti-β2-glycoprotein (Anti-B2GPI), anti-phosphatidylserine/prothrombin, and others, have been identified as potential causes of recurrent pregnancy loss (RPL) in women (9, 2). Although the exact causes of autoimmunity are varied, a growing body of experimental studies suggests that genetic predisposition combined with environmental factors through immune dysregulation can be responsible for autoantibody production in women with RPL (10).

In recent decades, vitamin D has received considerable attention as a crucial immunomodulatory factor in autoimmune diseases (11, 12). Several reports have shown that that autoimmune diseases are linked to vitamin D deficiency, which can impact both their severity and susceptibility (13). We found a high prevalence of vitamin D insufficiency and deficiency in pregnant women (14) and an association with certain negative pregnancy outcomes including RPL and preterm labor, when we examined the literature on vitamin D and pregnancy outcomes (15-17). 

The association between maternal immune dysregulation due to poor vitamin D status and the production of autoAbs has not been conclusively confirmed (18, 19). However, some studies have shown that low vitamin D levels are associated with the development of autoAbs in women with RPL (20, 21). The present study aimed to compare the serum vitamin D levels in RPL patients with or without autoantibodies and healthy women. We hypothesized that serum vitamin D concentration among RPL patients with autoAbs is lower than RPL patients without autoAbs.

## Methods

**Patients: **No pregnant women with a history of primary RPL who were referred to the clinic of Imam Reza Hospital in Bojnourd, Iran, between March and June 2021 were included in this study. Fifty-eight cases were positive for at least one autoantibody (ANA, anti-TPO, or APAs; aβ2GPI, LA, or aCL), and thirty-four cases were seronegative for autoAbs. A gynecologist, after clinical and paraclinical examination cases, confirmed RPL not resulting from endocrine, anatomic, or genetic abnormalities, and infection. The healthy control group included fifty-eight cases of no pregnant women who have formerly had at least one successful pregnancy without complications and no history of abortion or stillbirth. This control group was age-matched to the women with RPL and was negative for autoAbs (ANA, anti-TPO, or APAs; aβ2GPI, LA, or aCL). In the present study, the creative diagnostics ELISA kit (USA) was used to detect different autoAbs through indirect ELISA at 450 nm.

Demographic data and clinical characteristics of women with RPL and healthy controls, including age, region, religion, habits, physiological and reproductive history, etc. were collected with a questionnaire and a physical examination by a gynecologist. 

The study did not comprise women who had suffered from a previous miscarriage attributable to chromosomal abnormalities within the embryo, uterine anatomical anomalies, endocrine factors, or infections. We also excluded women who met any of the following criteria: systemic disease, hypertension, liver disease, malignancy, uncontrolled diabetes, previous venous or arterial thrombosis, chronic kidney disease, and rheumatic disease. The participants mentioned that they had been consuming either vitamin D supplements or drugs that hinder the absorption of vitamin D in the last three months.

We removed geographical or seasonal confounding factors by recruiting study subjects confined to women living in Bojnourd and referred to the clinic of Imam Reza Hospital in Bojnourd, Iran, from March to June 2021(spring months in Iran). The present inquiry was conducted in accordance with established ethical principles and guidelines, following the receipt of informed consent from all participants. Approbation for the study was obtained from the Ethics Committee of North Khorasan University of Medical Sciences, located in Bojnourd, Iran (reference number for ethical approval: IR. NKUMSREC1400.173). 

**Blood collection and measurement and assessment of 25(OH) D: **The peripheral venous blood was collected from all subjects. Blood samples for serum isolation were centrifuged at 2000×g and were stored at -80 ◦C until later. Quantifying 25(OH) D in serum performed using commercial sandwich ELISA kits (PishtazTeb, Iran) according to the manufacturer's recommendations. The ELISA exhibited intra-assay and inter-assay precision values of less than 10% and 12%, respectively, for 25(OH) D. Based on the findings of the prior investigation, the thresholds designated for vitamin D insufficiency and deficiency were established as 30ng/ml and 10ng/ml, respectively (22).

**Statistical analysis: **The statistical analysis of the data was performed using GraphPad Prism software, specifically Version 5. 0 provided by GraphPad Software, USA. Median and ranges or mean values and standard deviations were calculated for the continuous variables. The Kolmogorov-Smirnov test was utilized to evaluate the normality of the serum levels of the 25(OH) D variable. Continuous variables were subjected to comparison through t-tests or one-way analysis of variance (ANOVA), whereas categorical dependent data analysis was carried out using either χ2 tests or Fisher’s exact tests as appropriate. 

The data were represented in an academic manner by indicating the mean value along with the standard error of the mean (SEM). Values of p<0.05 (*) were considered significant. 

## Results

**Characteristics of patients:** The demographic and medical data of the study groups are compared in table 1. In this study, we did not find statistical differences between the study groups regarding age, BMI, and length of marriage (P˃0.05), while there was a significant difference between RPL patients with autoAbs and RPL patients without autoAbs in the member of previous pregnancy losses (P=0.01, table 1). 

**Figure 1:**
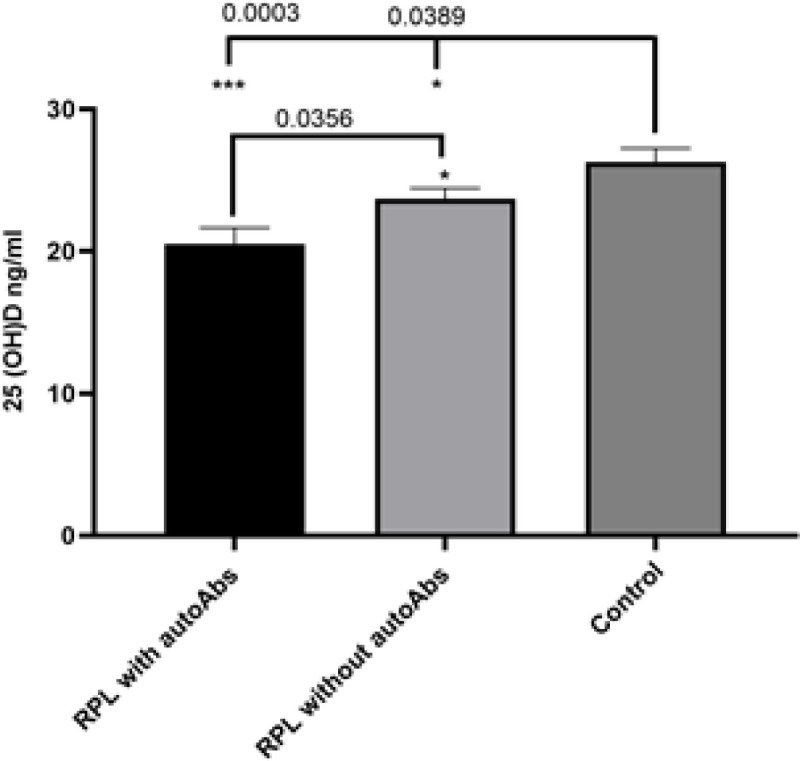
Comparison of serum levels of 25 (OH) D between the RPL patients with or without autoantibodies and healthy control group. There were significant differences between the study groups in serum levels of 25 (OH) D.Data represents mean ± SEM. *P < 0.05 was considered statistically significant. RPL: Recurrent pregnancy loss, autoAbs: Autoantibodies

**Measurement of 25(OH)D concentration in serum:** Determination of serum 25 (OH) D levels showed RPL patients and the healthy control group have insufficiency levels of it. The results of our study revealed a statistically significant decrease in 25 (OH) D serum concentrations among patients with RPL in comparison to women who were considered healthy controls (21.16 ± 1.24 vs. 25.04 ± 1.28,* P*=0.0006). Further analysis indicated that serum levels of 25 (OH) D in RPL patients with autoAbs were significantly lower than RPL patients without autoAbs (20.51 ± 1.15 ng/ml Vs. 23.69 ± 0.74 ng/ml, *P*=0.0356) (figure 1). 

**Type and frequency of autoantibodies detected in RPL patients:** In the present study, women with RPL had different types of autoAbs, including ANA, anti-TPO, LA, aCL, and anti-B2GPI. The anti-TPO was the most frequent of autoAbs in them. Surprisingly, multiple autoAbs (aCL, anti-B2GPI, and LA) were presented in an RPL case. Table 2 presents the type and frequency of autoAbs detected in RPL patients. 

**Figure 2:**
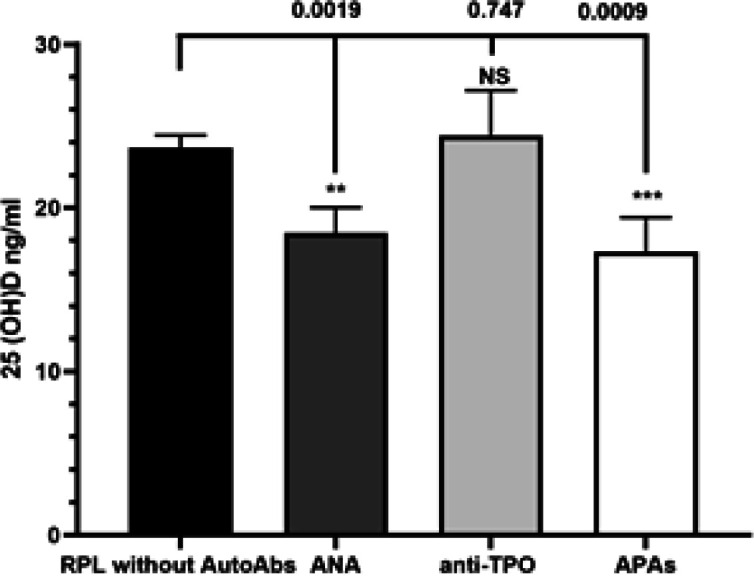
Comparison of serum levels of 25(OH) D between the RPL patients with different types of autoantibodies and RPL patients without autoantibodies. Data represents mean ± SEM. *P < 0.05 was considered statistically significant. Recurrent pregnancy loss, autoAbs: Autoantibodies; NS, non-significant; ANA, Antinuclear antibodies; anti-TPO, anti-thyroid peroxidase antibodies; APAs, Antiphospholipid antibodies; including lupus anticoagulant (LA), anti-cardiolipin (aCL), anti-β2-glycoprotein (Anti-B2GPI)

**Comparison of serum levels of 25 (OH) D between RPL patients with or without autoantibodies:** Our results, as shown in fig. 2, indicate that RPL patients with positive APAs and ANA have statistically lower than 25(OH)D serum levels compared with RPL patients without autoAbs (18.48 ± 1.53 and 17.32 ± 2.09 vs. 25.04 ± 1.28, *P*˂0.05). The concentration of 25 (OH) D were not significantly different in RPL women with positive anti-TPO and RPL women who did not have autoantibodies (24.43 ± 2.74 vs. 25.04 ± 1.28, *p*˃0.05) (figure2).

**Table1:** Demographic and clinical characteristics of RPL patients in comparison with control group. Data are the mean ± SEM. *P< 0.05 was considered statistically significant

	**RPL with AutoAbs**	**RPL** ^*^ ** Without AutoAbs** ^**^	**Control**	***P-*** **value**
**Age (years)**	30.96 ± 0.73	30.85 ± 0.83	29.21 ± 0.59	0.13
**BMI** ^***^ ** (Kg/m** ^2^ **)**	26.95± 0.85	27.40 ± 0.75	25.75 ± 0.52	0.13
**Length of marriage (years)**	8.62 ± 0.72	8.55 ± 0.89	7.97 ± 0.56	0.47
**Number of successful pregnancies**	Non	Non	1.83 ± 0.12	-
**Previous pregnancy loss**	3.03 ± 0.14	2.50 ± 0.09	Non	0.01

*Recurrent Pregnancy Loss, ^**^Autoantibodies, ^***^Body Mass Index

**Table 2:** Type and frequency of autoantibodies detected in the RPL patients

**Autoantibody**	**Frequency(Percentage)**
**Antinuclear antibodies (ANA)**	14 (24.1)
**Anti-thyroid peroxidase (anti-TPO)**	19 (32.8)
**Anti-cardiolipin (aCL)**	9 (15.5)
**Anti-β2-glycoprotein (anti-B2GPI)**	6 (10.3)
**lupus anticoagulant (LA)**	3 (5.2)
**ANA + anti-TPO**	3 (5.2)
**aCL + anti-TPO**	3 (5.2)
**LA + aCL+ anti-B2GPI**	1 (1.7)

## Discussion

The importance of vitamin D insufficiency or deficiency in relation to maternal immune dysregulation and its negative effects on pregnancy outcomes is supported by a growing body of evidence. However, whether its deficiency or insufficiency may be associated with autoantibody production in RPL patients has not been definitively determined. Thus, in this study, the vitamin D status of women with RPL who were seropositive for autoAbs was to be determined. 

The current study found that RPL patients with or without autoAbs have insufficiency levels of vitamin D, and their serum levels are lower than in healthy women. Following the present results, Goncalves*et al*. and Sharif *et al*., in the literature review, highlighted a significant proportion of women with recurrent pregnancy loss to have an insufficient or deficient level of vitamin D (14, 21). They recommended this might be related with immunological dysregulation and RPL (14, 21). Another important finding was that RPL patients who were positive for ANA and APAs, except anti-TPO, had markedly lower than vitamin D levels than RPL patients without autoAbs. This study supports evidence from previous observations; lower vitamin D levels were correlated with a higher incidence of ANA (23, 24). Prior research has also confirmed that decreased levels of vitamin D are more common in RPL patients who were seropositive for APAs (25). Therefore, our data are consistent with other studies which have shown a significant increase in the prevalence of APAs in women with RPL in the low vitamin D group compared to the normal vitamin D group. 

There are several possible explanations for this result. The presented findings can be elucidated through the observation that the regulation of B cells which relies on vitamin D is crucial in maintaining regular B cell homeostasis. When there is a deficiency of vitamin D, it paves the way for augmented B cell activity and the development of humoral autoimmunity (26). Another possible explanation is that vitamin D-mediated regulation of regulatory T cell functions during pregnancy (25). It has been reported that low serum concentration of vitamin D affects the activity and frequency of Treg cells in patients with RPL (27).Recently, Clement et al. have emphasized regulatory T cells by suppressing follicular T helper cells to prevent from restraining early B cell responses and autoantibody production(28). In confirmation of this, Jakiela*et al*. and Álvarez-Rodríguez *et al*. have indicated that the frequency of regulatory T cell decrease in patients with positive APAs or ANA (29, 30). It is now well-established that anti-TPO positivity during pregnancy is associated with pregnancy complications (31). The current investigation results did not indicate a correlation between the levels of serum 25(OH)D and anti-TPO.

This finding is in congruence with the research conducted by Goswami*et al*., wherein they observed a weak negative correlation between the levels of serum 25(OH)D and anti-TPO in individuals with autoimmune thyroid disease (32). In contrast to the findings of Shin et al., it was observed that females diagnosed with autoimmune thyroid exhibited reduced levels of 25(OH) D when compared to their healthy counterparts. In contrast to our data, they have suggested that a low concentration of 25(OH) D is associated with susceptibility to autoimmune thyroid disease (33). This inconsistency may be due to the limited number of RPL patients who were only positive for anti-TPO, not for other thyroid autoAbs (anti-thyroid-globulin antibody and TSH receptor autoAbs).In confirmation of this, Wang *et al*. have shown that there is a relationship between the titers of anti-thyroid-globulin antibody titers and the levels of serum 25(OH)D among females, but a weak association between anti-TPO titers and levels of serum 25(OH)D (34).

These findings suggest that RPL patients, especially RPL patients who were positive for APAs or ANA had low concentration of 25(OH)D compared to healthy women. These findings may provide insight into an association of maternal immune dysregulation due to insufficient vitamin D status and the production of autoAbs in RPL patients. Perhaps supplementation of vitamin D in patients with RPL who are positive for APAs or ANA is associated with a better pregnancy outcome. However, these findings may be somewhat limited by the single-center study design, small sample size, the method used to measure 25 (OH)D, limited types of autoAbs detected in study groups, and different times after pregnancy loss for blood sampling. Further investigation includes serum concentration of vitamin D-binding protein (VDBP), determination of vitamin D receptor (VDR) genetic polymorphisms, expression levels of vitamin D receptor at the maternal-fetal interface, and investigation of the frequency and function of regulatory T cells may help to develop a complete understanding of the correlation between vitamin D status and induction of autoantibody production in the patients with RPL. 
